# Evaluation of the major changes in eighth edition of the American Joint Committee on Cancer pathological staging for prostate cancer treated with prostatectomy

**DOI:** 10.1371/journal.pone.0187887

**Published:** 2017-11-09

**Authors:** Wen-jun Xiao, Yao Zhu, Bo Dai, Ding-wei Ye

**Affiliations:** 1 Department of Urology, Fudan University Shanghai Cancer Centre, Shanghai, People’s Republic of China; 2 Department of Oncology, Shanghai Medical College, Fudan University, Shanghai, People’s Republic of China; National Health Research Institutes, TAIWAN

## Abstract

This study aimed to evaluate the major changes of the eighth edition of the American Joint Committee on Cancer (AJCC) pathologic staging for prostate cancer treated with radical prostatectomy. A total of 138,176 patients diagnosed with prostate adenocarcinoma undergoing radical prostatectomy were selected from the Surveillance, Epidemiology and End Results (SEER) database during 2004–2014 period. Excluded were cases with incomplete or unavailable staging, PSA and Gleason score information. Two subgroups were established: group a, T2 stage with PSA≥20ng/ml; group b, T2 stage with Gleason score grade group 5 and PSA<20ng/ml. The median follow-up time was 58 months. The median age at diagnosis for the overall group was 61 years, and the median PSA was 5.7ng/ml. Cancer-specific survival (CSS) at tenth years was 99.3% for T2a/T2b, 99.2% for T2c, respectively. The survival differences between T2a/T2b and T2c did not have statistical significance (*P* = .323). It was necessary for the current eighth edition to define a single pathologic T2 category, eliminating the subcategories, for all organ-confined disease.CSS at the tenth years was 98.4% for group a, 92.6% for group b, respectively. The prognosis of group a was worse than AJCC II (*P* = .002). The prognosis of group b was not only worse than AJCC II (*P* < .001), but also worse than AJCC IIIB. There was necessity to separate the disease with PSA≥20ng/ml or Gleason score grade group 5 from other organ-confined disease. The present study supported the scientificity of the eighth edition of AJCC pathologic staging for prostate cancer.

## Introduction

The first uniform staging system for prostate cancer was published by the American Joint Committee on Cancer (AJCC) and the Union Internationale Contre Cancer (UICC) in 1992[1]. The AJCC has published eighth edition Cancer Staging Manual in 2017[2]. This new version for prostate cancer staging has been significantly modified. In this version, the prognostic roles of PSA and tumor grade are enhanced, and the newly proposed Gleason score grade grouping system is also used. National Cancer Institute Surveillance, Epidemiology and End Results (SEER)database collects and reports data from 17 population-based cancer registries representing about 28% of the United States population, and has recently completed the quality control of PSA data since 2004[3]. Therefore, this study intends to use SEER to evaluate the major changes of AJCC new edition for prostate cancer treated with radical prostatectomy.

## Materials and methods

The SEER*Stat 8.3.4 software (NCI, Silver Spring, MD) was queried to find all cases of men diagnosed with prostate adenocarcinoma undergoing radical prostatectomy between the years 2004 and 2014 [4].The International Classification of Disease for Oncology (ICD-O-3) site code C61.9 (prostate) and histology codes 8140 (adenocarcinomas) as well as site-specific surgery codes 50 were used to generate a cohort with pathologically based staging. Excluded cases were that with incomplete or unavailable staging, PSA or Gleason score information. According to the SEER research data record description of the present version submitted in November 2016, the follow-up cutoff date was December 31, 2014. The follow-up time was calculated from the diagnosis of the disease to the death or loss of the individual case, or to the cutoff date.

For each individual case listed after 2004, SEER provides AJCC TNM stages. If the cancer was confined to the prostate or if there was capsular invasion alone without extension through the capsule, this corresponds to stage pT2 disease per 8th AJCC criteria. Extension of cancer through the prostatic capsule and into periprostatic tissue, but without involvement of the seminal vesicles, was categorized as “extracapsular disease” (pT3a). Extension beyond the prostatic capsule with extension to the seminal vesicles was categorized as “seminal vesicle invasion” (pT3b). Extension to or fixation of adjacent structures other than seminal vesicles was characterized as “extended direct extension” (pT4). Involvement of regional nodes was categorized as “regional nodal disease” (pN1), and metastases to bone, distant soft tissue, other organs, and nonregional nodes were categorized as “metastatic disease” (M1) [2, 5].

Gleason score is provided in the SEER database through “CS site-specific factor”. The World Health Organization and the International Society for Urologic Pathology formalized changes to the Gleason system and adopted grade groups for prostate cancer [6]. As described in detail previously[5], the grade groups are now numbered from 1 to 5, where grade group 1 is similar to Gleason sum≤6 tumors, grade group 2 is similar to 3+4 = 7 tumors, grade group 3 is similar to 4+3 = 7 tumors; grade group 4 is similar to Gleason sum8 tumors, and grade group 5 is similar to Gleason sum 9and 10 tumors [6, 7].

The death due to prostate cancer was considered as the endpoint event in the present study. Cancer-specific survival (CCS) was defined as the time from cancer diagnosis until death due to prostate cancer, which was provided by the SEER cause-specific death classification variable. CCS was compared among different pathological stages using the Kaplan-Meier method and log-rank test. The subgroups for the each comparison were mutually exclusive. The proportional hazard assumption was checked to ensure it was not violated. All statistical analyses were conducted with R statistical software, version 2.15.2 (R Core Team, 2012, http://www.r-project.org) with add-on packages of Survival and Hmisc [8]. Statistical significance was set at P < .05. All tests were 2-tailed.

## Results

A total of 138,176 cases were identified from the 2004–2014 period using the aforementioned inclusion and exclusion criteria. Descriptive characteristics for the cohort were shown in Table 1. The median follow-up time was 58 months. The median age at diagnosis for the overall group was 61 years, and median PSA was 5.7ng/ml. White men comprised most cases (82%), followed next by African American men (12%). Approximately equal proportions of different ethnic groups were diagnosed within each AJCC stage.

**Table 1:** Demographic, clinical and pathologic characteristics of the cohort.

	I	IIA	IIB	IIC	IIIA	IIIB	IIIC	IV	All
**N**	43786	3890	35181	13938	2242	28970	5873	4296	138176
**Age at diagnosis (median, yr)**	60	62	61	63	62	62	64	62	61
**PSA (median, ng/ml)**	4.9	12.1	5.4	5.95	29.0	6.7	7.5	10.2	5.7
**Ethnicity**									
White	84%	78%	81%	79%	76%	82%	82%	82%	82%
Black	11%	15%	13%	14%	16%	12%	10%	13%	12%
Other	4%	6%	4%	7%	6%	5%	8%	5%	5%
unkown	1%	1%	1%	1%	1%	1%	1%	1%	1%
**T stage**									
T1	1%	1%		1%	1%				1%
T2a T2b	21%	23%	14%	17%	16%		6%	3%	13%
T2c	78%	75%	86%	82%	83%		25%	14%	60%
T3a						69%	33%	28%	17%
T3b						23%	29%	42%	7%
T4						7%	6%	12%	2%
**Gleason score group**									
1	100%	100%			37%	17%		5%	39%
2			100%		35%	43%		20%	36%
3				67%	17%	24%		22%	13%
4				33%	10%	15%		22%	7%
5							100%	32%	5%
**Vital status**									
Alive	97%	95%	96%	96%	95%	94%	88%	88%	95%
Dead	3%	5%	4%	4%	5%	6%	12%	12%	5%
due to Prostate/ due to all death	4%	4%	4%	11%	11%	80%	53%	57%	20%
due to other reasons/due to all death	96%	96%	96%	89%	89%	20%	47%	43%	80%

**Fig 1:**
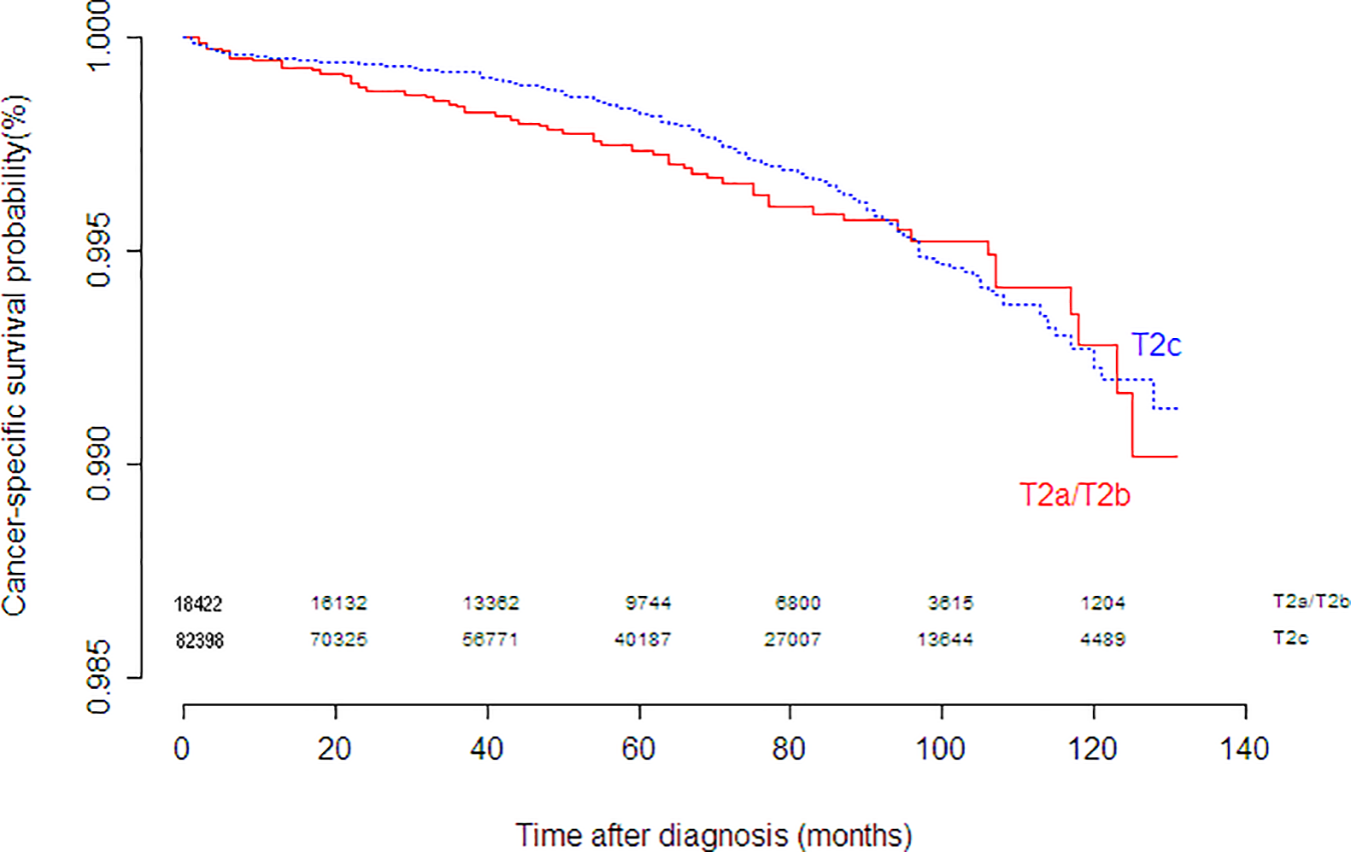
Kaplan-Meier curves for cancer-specific survival of T2a/T2b and T2c.

In the seventh edition, a 3-tier system was used to subdivide pathologic T2 disease (pT2) based on the extent and laterality of disease. The current eighth edition defines a single pT2 category, eliminating the subcategories, for all organ-confined disease. In the present SEER cohort, CSS at 10 years was 99.3% for T2a/T2b, 99.2% for T2c, respectively. The survival differences between T2a/T2b and T2c did not have statistical significance (*P* = .323), as depicted in Fig 1 and Table 2.

**Table 2:** Comparison of cancer-specific survival among different subgroups.

Stage	N	CSS at 10th year (95%CI)	Median follow-up time (month)	Hazard ratio (95%CI)	*P*
T2a/T2b	18422	0.993(0.990–0.995)	64	Reference	
T2c	82398	0.992(0.991–0.994)	58	0.867(0.652–1.151)	0.323
II	52690	0.992(0.990–0.994)	58	Reference	
T2& PSA≥20ng/ml (group a)	2220	0.984(0.974–0.993)	55	2.347(1.355–4.064)	0.002
T2& Gleason score group 5 & PSA<20ng/ml (group b)	1711	0.926(0.886–0.967)	53	Reference	
II	52690	0.992(0.990–0.994)	58	0.140(0.095–0.206)	<0.001
IIIB	28970	0.965(0.960–0.969)	55	0.623(0.432–0.900)	0.011

CSS: **Cancer-specific survival probability, CI: Confidential interval**

**Fig 2:**
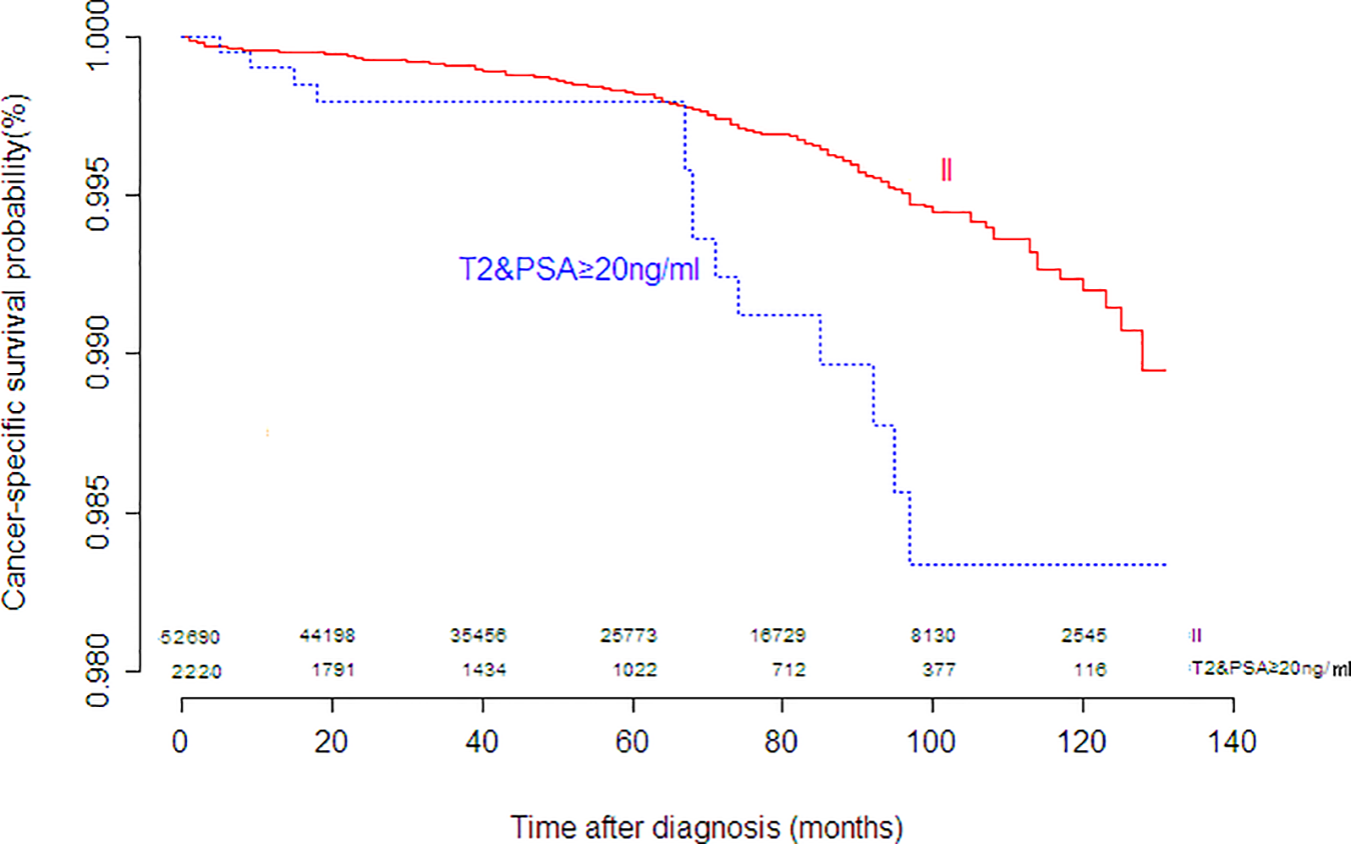
Kaplan-Meier curves for cancer-specific survival of stage II and T2 with PSA≥20ng/ml.

**Fig 3:**
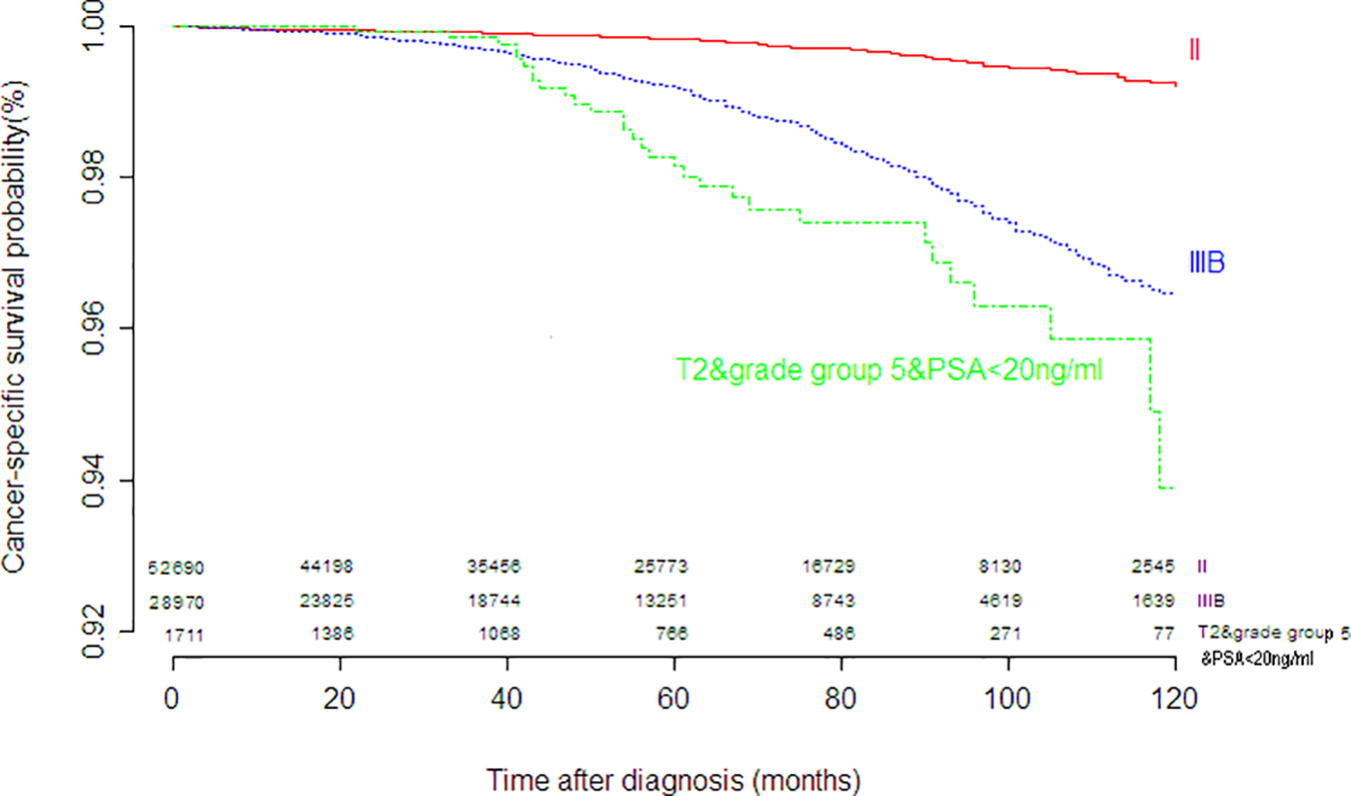
Kaplan-Meier curves for cancer-specific survival of stage II, stage IIIB and T2 with grade group 5 and PSA≥20ng/ml.

The other changes of the eighth edition were the separation of some organ-confined disease from stage II into stage III based upon the initial serum PSA level and the newly proposed Gleason score grade groups. To evaluate these changes, two subgroups were established: group a, T2 stage with PSA≥20ng/ml; group b, T2 stage with Gleason score grade group 5 and PSA<20ng/ml. CSS at the tenth years was 98.4% for group a and 92.6% for group b, respectively. The survival differences between AJCC II and group a had statistical significance (*P* = .002), as depicted in Fig 2 and Table 2.The prognosis of group b was not only worse than AJCC II (*P* < .001), but also worse than AJCC IIIB(*P* = .011), as depicted in Fig 3 and Table 2. These results reflected the necessities of separating organ-confined diseases with PSA≥20ng/ml or grade group 5from stage II.

## Discussion

This study examined a large population-based database to report cancer-specific survival outcomes of prostate cancer classified by different pathological stages. As generally acknowledged before, the overall cancer-specific mortality rates of the present cohort also remain very low and the median follow-up time of about 5 years may be not long enough for patients undergoing radical surgery. However, because of large scale and high data quality, this cohort can be used to evaluate the major changes of AJCC 8th edition for prostate cancer.

An important change in this edition is that pT2 is no longer subdivided. The substaging of pT2 prostate cancers has been a matter of debate given the reversal in the recent TNM systems as compared to the simplification of the system in 1997 [1]. A number of literatures demonstrated the lack of prognostic information of pathological substaging of T2 stage [7, 9].The present study also confirmed that there was no significant difference in CSS between pT2a/2b and pT2c. In this way, the pathological T staging of all localized prostate cancer is defined aspT2, bringing simplification to the staging system, while prognosis subdivision in this group depends mainly on the nonanatomic factors (grade groups and serum PSA).

The nonanatomic factors are important for the prognosis of prostate cancer. The existing risk classifications systems, such as D'amico and NCCN (National Comprehensive Cancer Network), have used these nonanatomic factors to place patients into different risk categories. The seventh edition of the AJCC staging manual has also considered the effect of grade and initial PSA on early-stage prostate cancer, whereas previous editions relied only on TNM staging without consideration of Gleason score and PSA level [10]. This revision has clearly improved the predictive value of the staging system for early-stage prostate cancer [11]. However, in the seventh edition, due to the complexity of the pT staging, the role of PSA and Gleason score for prognostic staging division is unsimplified and difficult to remember and apply.

For initial serum PSA, the eighth edition still uses the 10ng/ml and 20ng/ml as cutoff points. To sub-staging the patients with Gleason score sum ≤6, AJCC stage I is defined as PSA<10ng/ml, and AJCC stage IIA is defined as 10≤PSA<20ng/ml. The cases with PSA≥20ng/ml were classified to stage III. For tumor grade of prostate cancer, the eighth edition uses a new histopathologic classification system. This 5-tier system has divided the cases with Gleason score sum of 7 into different stages based upon the primary Gleason score. The cases with Gleason score sum of 7 occupied more than half of the whole cohort which were treated with radical prostatectomy. The division of such a large group has indicated its superior prognostic value in previous literature [12]. The sub-staging of eighth edition of AJCC stage group II mainly depended on grade groups, while the cases with grade group 5 were classified to stage III.

As mentioned earlier, for the eighth edition of AJCC, prognostic stage group III includes some organ-confined prostate cancers based on PSA and grade group status. According to the general principle of tumor staging, AJCC prognostic stage group III is always associated with nonorgan-confined disease. Although there has been stage III designation of organ-confined disease in other solid tumor types [13], it is the first time for prostate cancer to incorporate organ-confined disease in stage III. Disease with higher PSA level or higher tumor grade might perform worse than the subset with higher stage but lower PSA level or lower tumor grade. In this study, the prognosis of the cases with PSA≥20ng/ml was worse than that of the cases with stage II which consisted of cases with PSA<20ng/ml, while the prognosis of the grade group 5was not only worse than that of the AJJC stage II with the similar condition (PSA<20ng/ml and T2), but also worse than that of the AJCC stage IIIB. It is not appropriate for these subgroups to remain in the stage II. So in the eighth edition these subgroups are classified into stage IIIA and IIIC, respectively.

There are some limitations to this study. SEER database has its inherent shortcomings, such as coding errors, lack of detailed treatment information and recurrence information. In addition, because the quality control has not been completed, the PSA data before 2004 are not applicable. This results in a shorter follow-up time and a relatively small number of endpoint events.

## Conclusions

Our study supports the modification of AJCC pathological staging manual for prostate cancer. There is necessity of defining a single pT2 category and eliminating the subcategories, as well as separating the disease with PSA≥20ng/ml or grade group 5 from other organ-confined disease.
